# 2-(1*H*-Benzotriazol-1-yl)-1-(3-methyl­benzo­yl)ethyl benzoate

**DOI:** 10.1107/S1600536809026853

**Published:** 2009-07-18

**Authors:** Wu-Lan Zeng, Lian-Cai Du, Lei Zhang, Fang-Fang Jian

**Affiliations:** a MicroScale Science Institute, Department of Chemistry and Chemical Engineering, Weifang University, Weifang 261061, People’s Republic of China; b Microscale Science Institute, Department of Biological Engineering, Weifang University, Weifang 261061, People’s Republic of China

## Abstract

In the title mol­ecule, C_23_H_19_N_3_O_3_, the dihedral angles between the mean plane of the benzotriazole ring system and the benzene and phenyl rings are 9.67 (9) and 86.08 (10)°, respectively. The dihedral angle between the benzene and phenyl rings is 85.89 (11)°. In the crystal structure, weak inter­molecular C—H⋯O hydrogen bonds link mol­ecules into chains along [010].

## Related literature

For the pharmacological activities of 1*H*-benzotriazoles and their derivatives, see: Chen & Wu (2005 ▶). For standard bond-length data, see: Allen *et al.* (1987 ▶).
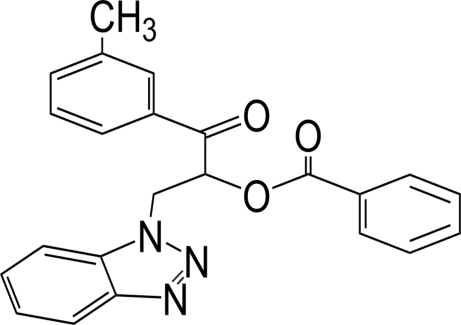


## Experimental

### 

#### Crystal data

C_23_H_19_N_3_O_3_
*M* *_r_* = 385.41
Monoclinic, 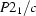

*a* = 10.1095 (5) Å
*b* = 9.3849 (4) Å
*c* = 20.7091 (10) Å
β = 99.061 (4)°
*V* = 1940.29 (16) Å^3^
*Z* = 4
Mo *K*α radiation
μ = 0.09 mm^−1^
*T* = 298 K
0.30 × 0.10 × 0.10 mm

#### Data collection

Siemens SMART CCD diffractometer
Absorption correction: multi-scan (*SADABS*; Sheldrick, 1996 ▶) *T*_min_ = 0.974, *T*_max_ = 0.991
16102 measured reflections
3301 independent reflections
2071 reflections with *I* > 2σ(*I*)
*R*_int_ = 0.051

#### Refinement

*R*[*F* ^2^ > 2σ(*F* ^2^)] = 0.044
*wR*(*F* ^2^) = 0.110
*S* = 1.00
3301 reflections
262 parameters
H-atom parameters constrained
Δρ_max_ = 0.19 e Å^−3^
Δρ_min_ = −0.22 e Å^−3^

### 

Data collection: *SMART* (Siemens, 1996 ▶); cell refinement: *SAINT* (Siemens, 1996 ▶); data reduction: *SAINT*; program(s) used to solve structure: *SHELXS97* (Sheldrick, 2008 ▶); program(s) used to refine structure: *SHELXL97* (Sheldrick, 2008 ▶); molecular graphics: *SHELXTL* (Sheldrick, 2008 ▶) and *PLATON* (Spek, 2009 ▶); software used to prepare material for publication: *SHELXTL*.

## Supplementary Material

Crystal structure: contains datablocks global, I. DOI: 10.1107/S1600536809026853/lh2841sup1.cif

Structure factors: contains datablocks I. DOI: 10.1107/S1600536809026853/lh2841Isup2.hkl

Additional supplementary materials:  crystallographic information; 3D view; checkCIF report

## Figures and Tables

**Table 1:** Hydrogen-bond geometry (Å, °)

*D*—H⋯*A*	*D*—H	H⋯*A*	*D*⋯*A*	*D*—H⋯*A*
C7—H7*B*⋯O2^i^	0.97	2.42	3.062 (2)	123
C11—H11*A*⋯O1^ii^	0.93	2.60	3.375 (2)	141

Symmetry codes: (i) 
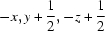
; (ii) 
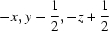
.

**Fig. 1.**
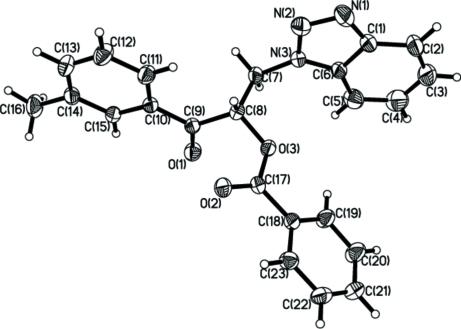
The molecular structure of (I), drawn with 30% probability ellipsoids.

**Fig. 2.**
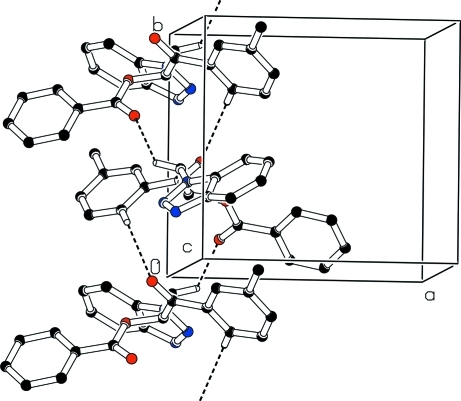
Part of the crystal structure with hydrogen bonds shown as dashed lines.

## supplementary crystallographic information

### Comment

1*H*-Benzotriazoles and their derivatives are an important class of compounds because they exhibit a broad spectrum of pharmacological activities such as antifungal, antitumor and antineoplastic activities (Chen & Wu., 2005). Herein, we present the crystal structure of the title compound (I). In (I) (Fig. 1) all bond lengths (Allen *et al.*, 1987) and angles within normal ranges. The benzotriazole ring system is essentially planar. The dihedral angles between the mean plane of the benzotriazole ring system and rings C10—C15 and C18—C23 are 9.67 (9) and 86.08 (10)°, respectively. The dihedral angle between rings C10—C15 and C19—C23 is 85.89 (11)°. In the crystal structure weak intermolecular C—H···O hydrogen bonds link molecules into chains along [010] (see Fig. 2).

### Experimental

Bromine (3.2 g,0.02 mol) was added dropwise to a solution of 3-(1*H*-benzo[*d*][1,2,3]triazol-1-yl)-1-*m*-tolylpropan-1-one (5.30 g,0.02 mol) and sodium acetate (1.6 g,0.02 mol) in acetic acid (50 ml). The reaction proceeded for 8 h. Water (50 ml) and chloroform (20 ml) were then added. The organic layer was washed successively with saturated sodium bicarbonate solution and brine, dried over anhydrou magnesium sulfate and the chloroform solution filtered. It was cooled with ice-water, and then an acetone solution (10 ml) of benzoic acid (2.24 g,0.02 mol) and triethylamine (2.8 ml) was added. The mixture was stirred with ice-water for about 6 h. The solution was then filtered and concentrated. Single crystals were obtained by slow evaporation of an acetone-ethylacetate (1:1 *v*/*v*) solution of (I) at room temperature over a period of one week.

### Refinement

All H atoms were located in difference Fourier maps and were subsequently constrained to ride on their parent atoms, with C—H distances in the range 0.93–0.98 Å, and with *U*_iso_(H) = 1.2 *U*_eq_(C) or 1.5 *U*_eq_(methyl C) H atoms.

### Crystal data

**Table:**

C_23_H_19_N_3_O_3_	*F*(000) = 808
*M**_r_* = 385.41	*D*_x_ = 1.319 Mg m^−^^3^
Monoclinic, *P*2_1_/*c*	Mo *K*α radiation, λ = 0.71073 Å
Hall symbol: -P 2ybc	Cell parameters from 3301 reflections
*a* = 10.1095 (5) Å	θ = 2.0–25.0°
*b* = 9.3849 (4) Å	µ = 0.09 mm^−^^1^
*c* = 20.7091 (10) Å	*T* = 298 K
β = 99.061 (4)°	Block, colorless
*V* = 1940.29 (16) Å^3^	0.30 × 0.10 × 0.10 mm
*Z* = 4	

### Data collection

**Table:**

Siemens SMART CCD diffractometer	3301 independent reflections
Radiation source: fine-focus sealed tube	2071 reflections with *I* > 2σ(*I*)
graphite	*R*_int_ = 0.051
φ and ω scans	θ_max_ = 25.0°, θ_min_ = 2.0°
Absorption correction: multi-scan (*SADABS*; Sheldrick, 1996)	*h* = −11→11
*T*_min_ = 0.974, *T*_max_ = 0.991	*k* = −11→11
16102 measured reflections	*l* = −23→24

### Refinement

**Table:**

Refinement on *F*^2^	Primary atom site location: structure-invariant direct methods
Least-squares matrix: full	Secondary atom site location: difference Fourier map
*R*[*F*^2^ > 2σ(*F*^2^)] = 0.044	Hydrogen site location: inferred from neighbouring sites
*wR*(*F*^2^) = 0.110	H-atom parameters constrained
*S* = 1.00	*w* = 1/[σ^2^(*F*_o_^2^) + (0.0483*P*)^2^ + 0.2024*P*] where *P* = (*F*_o_^2^ + 2*F*_c_^2^)/3
3301 reflections	(Δ/σ)_max_ < 0.001
262 parameters	Δρ_max_ = 0.19 e Å^−^^3^
0 restraints	Δρ_min_ = −0.22 e Å^−^^3^

### Special details

**Table:**

Geometry. All e.s.d.'s (except the e.s.d. in the dihedral angle between two l.s. planes) are estimated using the full covariance matrix. The cell e.s.d.'s are taken into account individually in the estimation of e.s.d.'s in distances, angles and torsion angles; correlations between e.s.d.'s in cell parameters are only used when they are defined by crystal symmetry. An approximate (isotropic) treatment of cell e.s.d.'s is used for estimating e.s.d.'s involving l.s. planes.
Refinement. Refinement of *F*^2^ against ALL reflections. The weighted *R*-factor *wR* and goodness of fit *S* are based on *F*^2^, conventional *R*-factors *R* are based on *F*, with *F* set to zero for negative *F*^2^. The threshold expression of *F*^2^ > σ(*F*^2^) is used only for calculating *R*-factors(gt) *etc*. and is not relevant to the choice of reflections for refinement. *R*-factors based on *F*^2^ are statistically about twice as large as those based on *F*, and *R*- factors based on ALL data will be even larger.

### Fractional atomic coordinates and isotropic or equivalent isotropic displacement parameters (Å^2^)

**Table:**

	*x*	*y*	*z*	*U*_iso_*/*U*_eq_	
O1	0.09785 (14)	0.45987 (15)	0.21346 (7)	0.0543 (4)	
O2	0.17138 (13)	0.14124 (15)	0.23494 (7)	0.0508 (4)	
O3	0.18369 (12)	0.29378 (14)	0.31866 (6)	0.0444 (4)	
C1	0.0844 (2)	0.2945 (3)	0.51412 (11)	0.0562 (6)	
C2	0.1674 (3)	0.2855 (3)	0.57479 (12)	0.0757 (8)	
H2B	0.1432	0.2312	0.6086	0.091*	
C3	0.2831 (3)	0.3582 (4)	0.58230 (14)	0.0886 (10)	
H3A	0.3400	0.3535	0.6222	0.106*	
C4	0.3204 (3)	0.4402 (3)	0.53236 (16)	0.0884 (9)	
H4A	0.4022	0.4876	0.5397	0.106*	
C5	0.2409 (3)	0.4538 (3)	0.47244 (13)	0.0677 (7)	
H5A	0.2654	0.5103	0.4394	0.081*	
C6	0.1216 (2)	0.3781 (2)	0.46443 (10)	0.0464 (5)	
C7	−0.0004 (2)	0.4194 (2)	0.34864 (9)	0.0518 (6)	
H7A	0.0510	0.5066	0.3485	0.062*	
H7B	−0.0943	0.4431	0.3358	0.062*	
C8	0.04164 (19)	0.3168 (2)	0.29917 (9)	0.0431 (5)	
H8A	−0.0068	0.2265	0.3000	0.052*	
C9	0.0140 (2)	0.3818 (2)	0.23043 (9)	0.0402 (5)	
C10	−0.11527 (19)	0.3540 (2)	0.18760 (9)	0.0391 (5)	
C11	−0.2093 (2)	0.2566 (2)	0.20324 (11)	0.0521 (6)	
H11A	−0.1922	0.2043	0.2418	0.062*	
C12	−0.3285 (2)	0.2383 (3)	0.16091 (12)	0.0634 (7)	
H12A	−0.3912	0.1729	0.1711	0.076*	
C13	−0.3551 (2)	0.3158 (2)	0.10391 (11)	0.0586 (6)	
H13A	−0.4359	0.3025	0.0762	0.070*	
C14	−0.2632 (2)	0.4134 (2)	0.08716 (10)	0.0479 (5)	
C15	−0.1438 (2)	0.4296 (2)	0.12935 (10)	0.0442 (5)	
H15A	−0.0804	0.4932	0.1184	0.053*	
C16	−0.2919 (3)	0.4998 (3)	0.02543 (11)	0.0743 (8)	
H16A	−0.3786	0.4747	0.0022	0.112*	
H16B	−0.2250	0.4804	−0.0015	0.112*	
H16C	−0.2905	0.5993	0.0362	0.112*	
C17	0.2376 (2)	0.1978 (2)	0.28153 (10)	0.0395 (5)	
C18	0.38089 (19)	0.1709 (2)	0.30448 (9)	0.0412 (5)	
C19	0.4567 (2)	0.2509 (2)	0.35264 (11)	0.0599 (7)	
H19A	0.4178	0.3263	0.3720	0.072*	
C20	0.5906 (2)	0.2187 (3)	0.37213 (12)	0.0713 (8)	
H20A	0.6414	0.2733	0.4044	0.086*	
C21	0.6490 (2)	0.1080 (3)	0.34462 (12)	0.0637 (7)	
H21A	0.7390	0.0871	0.3581	0.076*	
C22	0.5745 (2)	0.0282 (3)	0.29719 (12)	0.0604 (6)	
H22A	0.6140	−0.0479	0.2787	0.072*	
C23	0.4409 (2)	0.0590 (2)	0.27624 (11)	0.0514 (6)	
H23A	0.3915	0.0049	0.2433	0.062*	
N1	−0.0359 (2)	0.2318 (2)	0.49227 (10)	0.0793 (7)	
N2	−0.07420 (19)	0.2723 (2)	0.43222 (10)	0.0730 (6)	
N3	0.01911 (17)	0.36109 (19)	0.41419 (8)	0.0487 (5)	

### Atomic displacement parameters (Å^2^)

**Table:**

	*U*^11^	*U*^22^	*U*^33^	*U*^12^	*U*^13^	*U*^23^
O1	0.0460 (9)	0.0623 (10)	0.0531 (10)	−0.0116 (8)	0.0031 (7)	0.0094 (8)
O2	0.0483 (9)	0.0567 (9)	0.0447 (9)	−0.0043 (7)	−0.0009 (7)	−0.0106 (8)
O3	0.0398 (8)	0.0517 (9)	0.0394 (8)	0.0079 (7)	−0.0005 (6)	−0.0068 (7)
C1	0.0569 (15)	0.0742 (17)	0.0380 (14)	0.0125 (13)	0.0090 (12)	0.0009 (12)
C2	0.085 (2)	0.102 (2)	0.0395 (15)	0.0252 (18)	0.0090 (14)	0.0056 (14)
C3	0.078 (2)	0.136 (3)	0.0470 (18)	0.021 (2)	−0.0036 (16)	−0.0203 (18)
C4	0.0663 (18)	0.119 (3)	0.076 (2)	−0.0130 (17)	−0.0001 (17)	−0.040 (2)
C5	0.0687 (17)	0.0741 (17)	0.0603 (17)	−0.0128 (14)	0.0101 (14)	−0.0133 (14)
C6	0.0483 (13)	0.0536 (14)	0.0371 (13)	0.0106 (11)	0.0059 (11)	−0.0030 (11)
C7	0.0539 (14)	0.0633 (15)	0.0375 (13)	0.0198 (11)	0.0047 (11)	0.0069 (11)
C8	0.0360 (12)	0.0521 (13)	0.0397 (13)	0.0054 (10)	0.0008 (9)	0.0040 (10)
C9	0.0402 (12)	0.0422 (12)	0.0382 (12)	0.0050 (10)	0.0055 (10)	−0.0008 (10)
C10	0.0384 (12)	0.0415 (12)	0.0371 (12)	0.0015 (9)	0.0046 (9)	−0.0018 (10)
C11	0.0444 (13)	0.0583 (14)	0.0517 (14)	−0.0003 (11)	0.0021 (11)	0.0108 (11)
C12	0.0461 (14)	0.0701 (17)	0.0704 (18)	−0.0134 (12)	−0.0017 (12)	0.0155 (14)
C13	0.0472 (14)	0.0683 (16)	0.0547 (15)	−0.0041 (12)	−0.0090 (11)	−0.0019 (13)
C14	0.0500 (14)	0.0533 (14)	0.0389 (13)	0.0071 (11)	0.0021 (11)	−0.0007 (10)
C15	0.0403 (12)	0.0511 (14)	0.0405 (13)	−0.0014 (10)	0.0039 (10)	0.0014 (10)
C16	0.0744 (18)	0.092 (2)	0.0504 (15)	0.0029 (14)	−0.0092 (13)	0.0170 (14)
C17	0.0450 (13)	0.0398 (12)	0.0332 (12)	−0.0006 (10)	0.0045 (10)	0.0024 (10)
C18	0.0416 (12)	0.0432 (12)	0.0391 (12)	0.0001 (10)	0.0072 (10)	0.0032 (10)
C19	0.0476 (14)	0.0673 (16)	0.0611 (16)	0.0089 (12)	−0.0033 (12)	−0.0190 (13)
C20	0.0485 (15)	0.0864 (19)	0.0734 (18)	0.0059 (14)	−0.0077 (13)	−0.0231 (15)
C21	0.0421 (14)	0.0818 (18)	0.0669 (17)	0.0119 (13)	0.0079 (13)	0.0018 (15)
C22	0.0508 (15)	0.0639 (16)	0.0683 (17)	0.0132 (12)	0.0151 (13)	−0.0050 (13)
C23	0.0490 (14)	0.0518 (14)	0.0538 (14)	0.0006 (11)	0.0094 (11)	−0.0057 (11)
N1	0.0728 (15)	0.1137 (19)	0.0527 (14)	−0.0127 (14)	0.0142 (12)	0.0184 (13)
N2	0.0516 (12)	0.1129 (18)	0.0558 (14)	−0.0102 (12)	0.0118 (10)	0.0085 (13)
N3	0.0431 (11)	0.0660 (13)	0.0367 (11)	0.0073 (9)	0.0057 (9)	0.0051 (9)

### Geometric parameters (Å, °)

**Table:**

O1—C9	1.213 (2)	C11—H11A	0.9300
O2—C17	1.207 (2)	C12—C13	1.377 (3)
O3—C17	1.353 (2)	C12—H12A	0.9300
O3—C8	1.445 (2)	C13—C14	1.387 (3)
C1—N1	1.363 (3)	C13—H13A	0.9300
C1—C6	1.392 (3)	C14—C15	1.382 (3)
C1—C2	1.399 (3)	C14—C16	1.503 (3)
C2—C3	1.342 (4)	C15—H15A	0.9300
C2—H2B	0.9300	C16—H16A	0.9600
C3—C4	1.388 (4)	C16—H16B	0.9600
C3—H3A	0.9300	C16—H16C	0.9600
C4—C5	1.374 (4)	C17—C18	1.474 (3)
C4—H4A	0.9300	C18—C19	1.380 (3)
C5—C6	1.387 (3)	C18—C23	1.387 (3)
C5—H5A	0.9300	C19—C20	1.384 (3)
C6—N3	1.357 (2)	C19—H19A	0.9300
C7—N3	1.448 (2)	C20—C21	1.363 (3)
C7—C8	1.515 (3)	C20—H20A	0.9300
C7—H7A	0.9700	C21—C22	1.364 (3)
C7—H7B	0.9700	C21—H21A	0.9300
C8—C9	1.534 (3)	C22—C23	1.382 (3)
C8—H8A	0.9800	C22—H22A	0.9300
C9—C10	1.482 (3)	C23—H23A	0.9300
C10—C15	1.390 (3)	N1—N2	1.300 (3)
C10—C11	1.393 (3)	N2—N3	1.355 (2)
C11—C12	1.385 (3)		
			
?···?	?		
			
C17—O3—C8	114.33 (15)	C11—C12—H12A	119.7
N1—C1—C6	109.2 (2)	C12—C13—C14	121.0 (2)
N1—C1—C2	130.5 (2)	C12—C13—H13A	119.5
C6—C1—C2	120.3 (2)	C14—C13—H13A	119.5
C3—C2—C1	117.5 (3)	C15—C14—C13	117.8 (2)
C3—C2—H2B	121.3	C15—C14—C16	120.7 (2)
C1—C2—H2B	121.3	C13—C14—C16	121.4 (2)
C2—C3—C4	122.0 (3)	C14—C15—C10	122.27 (19)
C2—C3—H3A	119.0	C14—C15—H15A	118.9
C4—C3—H3A	119.0	C10—C15—H15A	118.9
C5—C4—C3	122.4 (3)	C14—C16—H16A	109.5
C5—C4—H4A	118.8	C14—C16—H16B	109.5
C3—C4—H4A	118.8	H16A—C16—H16B	109.5
C4—C5—C6	115.8 (2)	C14—C16—H16C	109.5
C4—C5—H5A	122.1	H16A—C16—H16C	109.5
C6—C5—H5A	122.1	H16B—C16—H16C	109.5
N3—C6—C5	134.1 (2)	O2—C17—O3	121.65 (18)
N3—C6—C1	103.80 (19)	O2—C17—C18	125.15 (19)
C5—C6—C1	122.1 (2)	O3—C17—C18	113.20 (17)
N3—C7—C8	112.51 (16)	C19—C18—C23	119.12 (19)
N3—C7—H7A	109.1	C19—C18—C17	123.06 (19)
C8—C7—H7A	109.1	C23—C18—C17	117.82 (18)
N3—C7—H7B	109.1	C18—C19—C20	119.9 (2)
C8—C7—H7B	109.1	C18—C19—H19A	120.1
H7A—C7—H7B	107.8	C20—C19—H19A	120.1
O3—C8—C7	106.18 (16)	C21—C20—C19	120.8 (2)
O3—C8—C9	110.28 (15)	C21—C20—H20A	119.6
C7—C8—C9	110.21 (16)	C19—C20—H20A	119.6
O3—C8—H8A	110.0	C22—C21—C20	119.5 (2)
C7—C8—H8A	110.0	C22—C21—H21A	120.2
C9—C8—H8A	110.0	C20—C21—H21A	120.2
O1—C9—C10	121.57 (18)	C21—C22—C23	120.9 (2)
O1—C9—C8	118.38 (18)	C21—C22—H22A	119.6
C10—C9—C8	119.99 (18)	C23—C22—H22A	119.6
C15—C10—C11	118.72 (19)	C22—C23—C18	119.8 (2)
C15—C10—C9	118.17 (18)	C22—C23—H23A	120.1
C11—C10—C9	123.11 (18)	C18—C23—H23A	120.1
C12—C11—C10	119.5 (2)	N2—N1—C1	107.9 (2)
C12—C11—H11A	120.3	N1—N2—N3	109.15 (19)
C10—C11—H11A	120.3	C6—N3—N2	109.98 (17)
C13—C12—C11	120.7 (2)	C6—N3—C7	130.49 (19)
C13—C12—H12A	119.7	N2—N3—C7	119.52 (18)

### Hydrogen-bond geometry (Å, °)

**Table:**

*D*—H···*A*	*D*—H	H···*A*	*D*···*A*	*D*—H···*A*
C7—H7B···O2^i^	0.97	2.42	3.062 (2)	123
C11—H11A···O1^ii^	0.93	2.60	3.375 (2)	141

Symmetry codes: (i) −*x*, *y*+1/2, −*z*+1/2; (ii) −*x*, *y*−1/2, −*z*+1/2.
